# Hepatic Remote Ischemic Preconditioning (RIPC) Protects Heart Damages Induced by Ischemia Reperfusion Injury in Mice

**DOI:** 10.3389/fphys.2021.713564

**Published:** 2021-10-04

**Authors:** Yanlong Ren, Shujin Lin, Wenxian Liu, Huiguo Ding

**Affiliations:** ^1^Department of Cardiology, Beijing Anzhen Hospital, Beijing Lab for Cardiovascular Precision Medicine, Capital Medical University, Beijing, China; ^2^College of Biological Science and Engineering, Fuzhou University, Fuzhou, China; ^3^Department of Gastroenterology and Hepatology, Beijing Youan Hospital, Capital Medical University, Beijing, China

**Keywords:** remote ischemic preconditioning, hepatic, ischemia reperfusion injury, left ventricular developed pressure, heart damage

## Abstract

It has been convincingly demonstrated that remote ischemic preconditioning (RIPC) can make the myocardium resistant to the subsequent ischemia reperfusion injury (IRI), which causes severe damages by mainly generating cell death. However, the cardioprotective effects of the hepatic RIPC, which is the largest metabolic organ against I/R, have not been fully studied. The aim of our research is whether remote liver RIPC may provide cardioprotective effects against the I/R-induced injury. Here, we generated an I/R mice model in four groups to analyze the effect. The control group is the isolated hearts with 140-min perfusion. I/R group added ischemia in 30 min following 90-min reperfusion. The third group (sham) was subjected to the same procedure as the latter group. The animals in the fourth group selected as the treatment group, underwent a hepatic RIPC by three cycles of 5-min occlusion of the portal triad and then followed by induction of I/R in the isolated heart. The level of myocardial infarction and the preventive effects of RIPC were assessed by pathological characteristics, namely, infarct, enzyme releases, pressure, and cardiac mechanical activity. Subjected to I/R, the hepatic RIPC minimized the infarct size (17.7 ± 4.96 vs. 50.06 ± 5, *p* < 0.001) and improved the left ventricular-developed pressure (from 47.42 ± 6.27 to 91.62 ± 5.22 mmHg) and the mechanical activity. Release of phosphocreatine kinase-myocardial band (73.86 ± 1.95 vs. 25.93 ± 0.66 IUL^−1^) and lactate dehydrogenase (299.01 ± 10.7 vs. 152.3 ± 16.7 IUL^−1^) was also decreased in the RIPC-treated group. These results demonstrate the cardioprotective effects of the hepatic remote preconditioning against the injury caused by I/R in the isolated perfused hearts.

## Introduction

Acute myocardial ischemia accounts for the most common cause of hospitalization in Western countries. Myocardial infarction is recognized as a heart attack when myocardial ischemia exceeds a threshold, which causes irreversible cardiac cell damage or even death (Yang et al., 2002). This perioperative myocardial infarction is the most dangerous following different non-cardiac surgeries, namely, liver transplantation (Devereaux et al., 2005; Polido et al., 2007). In the classical procedure, the reperfusion of coronary vessels by the primary percutaneous coronary intervention (PPCI) or thrombolytic therapy is the only way to reduce the myocardial injury (Keeley et al., 2003). Many studies indicate that reperfusion is accompanied by a detrimental manifestation known as reperfusion injury. The reperfusion injury commenced by the restoration of blood flow in the ischemic tissues may lead to additional injury and cell death that are reversibly damaged (Piper et al., 1998). Different intervention procedures have been suggested to reduce the damage induced by ischemia/reperfusion (I/R), wherein remote ischemic preconditioning (RIPC) is the latest one.

There is evidence that the myocardial injury induced by I/R can be significantly decreased by exposing the heart to nonlethal myocardial ischemia and reperfusion. This procedure is coined the term ischemic preconditioning (IPC) (Murry et al., 1986). This effect has also been observed in non-cardiac tissues with ischemia (Mounsey et al., 1992; Glazier et al., 1994; Roth et al., 1998; Yoshizumi et al., 1998), revealed that there is an innate protective mechanism against I/R injury. Since the implementation of IPC necessitates an invasive procedure to be applied directly to the myocardium, this may be impractical and/or even harmful in some clinical conditions. An alternative strategy is to induce the preconditioning *via* a position far from the heart. This approach referred as RIPC, was reported by Przyklenk et al. and extended to wide fields (Przyklenk et al., 1993; Donato et al., 2021). In fact, RIPC may represent a novel and inexpensive procedure to reduce perioperative ischemia complications (Kanoria et al., 2006; Kharbanda et al., 2006).

Although the underlying mechanisms of RIPC have not been yet fully understood, it is obvious that signaling may be transmitted *via* humoral (Dickson et al., 1999; Yang et al., 2019) or neural (Abdul-Ghani et al., 2017; Aulakh et al., 2017) pathway. K_ATP_ channels may play a pivotal function in this procedure. These channels are believed to be activated *via* protein kinase C (Cole et al., 1991; Garlid et al., 1997; Liang, 1997; Foster and Coetzee, 2016). It has been shown that IPC may protect the liver against ischemia-induced injury in different clinical conditions, namely, liver transplantation (Rampes and Ma, 2019). Lloris-Carsi et al. reported that the use of the IPC model increased the survival rate and reduced the levels of liver enzymes in mice (Lloris-Carsi et al., 1993). The data reported by other experimental studies indicate that transient hepatic ischemia can induce the protective effects against I/R injury in mice (Jin et al., 2010; Robertson et al., 2017). However, there are a few reports of applying the hepatic RIPC to protect the myocardium against the injury (Yang et al., 2017). Here, we hypothesized that remote liver IPC may provide cardioprotective effects against the I/R-induced injury in the same way that skeletal muscles, mesentery, and the kidney is shown (Donato et al., 2021).

## Materials and Methods

### Animals

Male FVB mice were purchased from the SPF animal center (Beijing, China). The FVB mice were kept in a room with controlled temperature and lighting (alternating 12 h periods of light and dark). All animals had free access to chow and water. All protocols on animals were approved by the Ethics Committee of Beijing Anzhen Hospital (approval number: 2018040X), Capital Medical University. All experimental procedures were carried out following the Guide for the Care and Use of Laboratory Animals published by the U.S. National Institutes of Health (publication, 8th Edition, 2011).

### The Surgery Procedure

The mice were anesthetized by 50 mg/kg thiopental sodium and then 250 IU heparin was injected *via* a ventral penile vein. A small incision was made in the midline of the abdomen to expose the liver. The procedure of hepatic RIPC was performed by a three-cycle of 5 min before establishing the I/R in the isolated perfused heart. To prepare the isolated rat heart, the heart was excised and the aorta was cannulated for anterograde perfusion. The non-recirculating Krebs–Henseleit buffer solution was used at a flow rate of 10 ml/min. The buffer solution is saturated with the gas mixture of 95% O_2_ plus 5% CO_2_ (pH = 7.4) and kept at a temperature of 37°C. A pressure transducer (MLT844 Physiological Pressure Transducer) was used to detect the coronary perfusion pressure. All isolated hearts were subjected to 30-min ligation of the left anterior descending coronary artery, followed by 90 of reperfusion, except for the control group.

### Experimental Protocol

**Figure 1:**
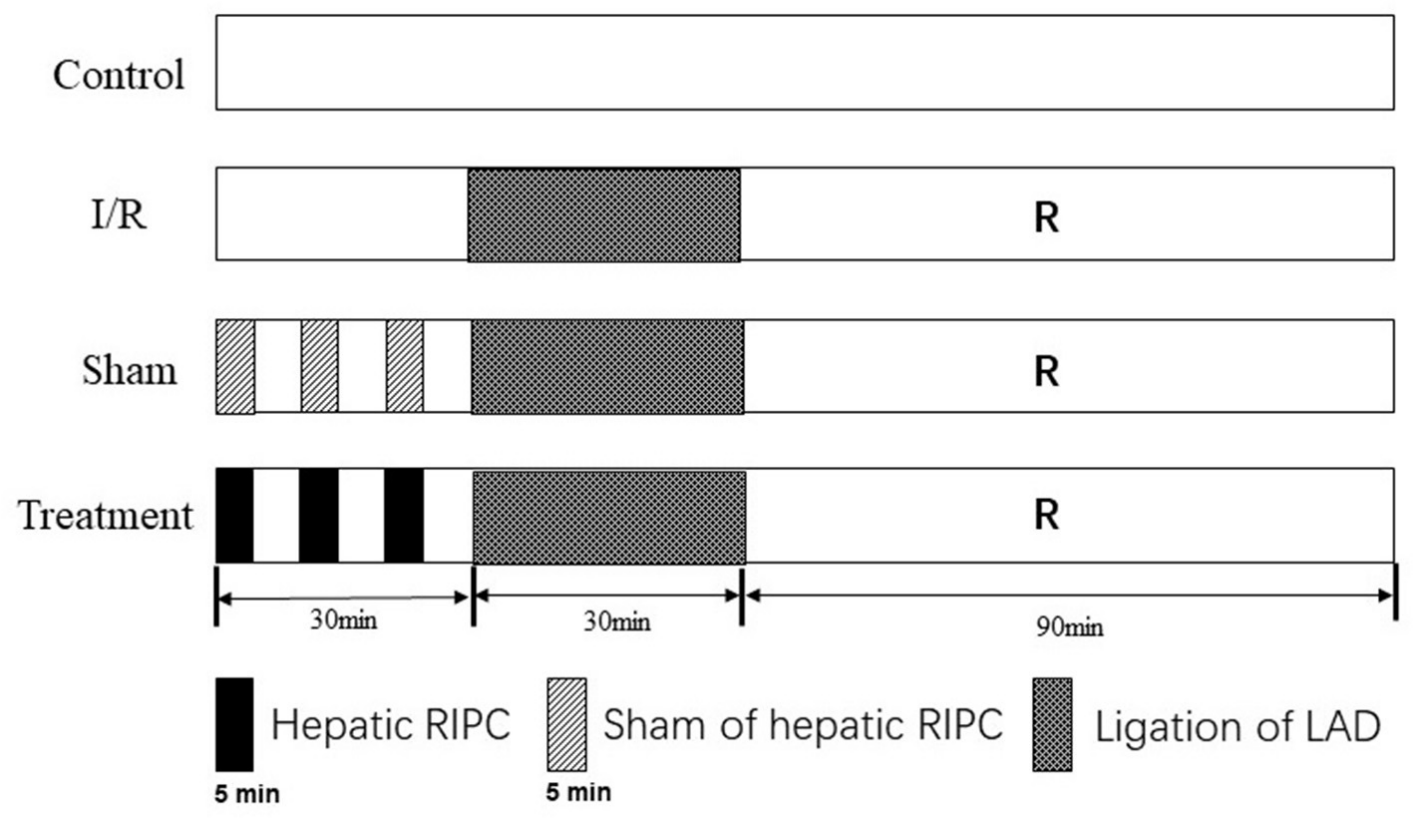
Experimental protocols. The procedure of hepatic RIPC was performed by a three-cycle of 5 min before establishing the I/R in the isolated perfused heart. All isolated hearts were subjected to 30-min ligation of the left anterior descending coronary artery, followed by 90 of reperfusion, except for the control group. In the control group, the Langendorff isolated heart perfusion was established in 150 min. I/R, ischemia/reperfusion; RIPC, remote ischemic preconditioning.

A total of 24 mice were randomly divided into four experimental groups. In the control group, the Langendorff isolated heart perfusion was established in 150 min. However, in the I/R group, the isolated hearts were exposed to ischemia for 30 min and then reperfusion for 90 min. The procedure conducted on the third (sham-operated) group was the same procedure as the I/R group except that a sham operation on the liver. The animals in the fourth group underwent a remote liver IPC by three cycles of 5-min occlusion of the portal triad before establishing the I/R in the isolated perfused heart (Figure 1).

The coronary effluent samples were collected respectively before ischemia and after 0, 5, 30, and 60 min of reperfusion. The collected samples were stored at −20°C until analyzing to measure the level of creatine kinase-myocardial band (CK-MB) and lactate dehydrogenase (LDH). Enzyme-linked immunosorbent assay kits were used for the analysis: serum CK-MB (Xinqidi, Wuhan, China) and serum LDH (X-Y Biotechnology, Shanghai, China).

### Hemodynamic Assay

Left ventricular-developed pressure (LVDP) was determined *via* an elastic water-filled balloon connected to a pressure transducer (MLT844 Physiological Pressure Transducer). The initial end-diastolic pressure was adjusted to 10 mmHg and after that, the pressure signals were recorded for analysis using Chart 5.0.1 Data Recording Software (PowerLab, ADInstruments Inc.). The heart rate, LVDP, and the percentage of the rate pressure produce (RPP%) were calculated from basic measurements.

### Myocardial Injury Assessment

At the end of the procedure of each experiment group, the heart was collected from the perfusion apparatus, and then it was sectioned from the apex to the base into 2-mm-thick slices. The infarct size, the area at risk of ischemic injuries, and the intensity of the I/R-induced myocardial injury were quantified using standard methods.

### Statistics

Significant differences were analyzed by the one-way ANOVA to compare between the experimental groups. A *p*-value of at least <0.05 was considered significant.

## Results

**Figure 2:**
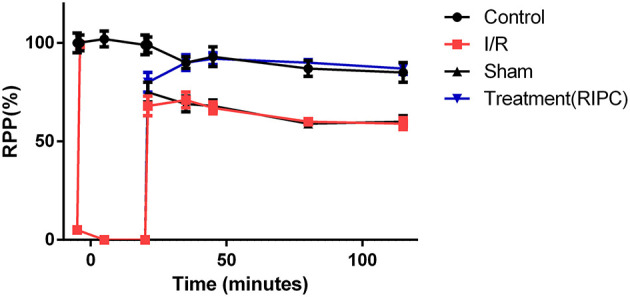
The effects of liver remote ischemic preconditioning on the rate pressure produce (RPP = HR × LVDP, mmHg beats min^−1^) in the isolated perfused rat hearts subjected to 30-min global ischemia followed by 90-min reperfusion. Values are expressed as means ± SEM obtained from at least six tested mice in each group. HR, heart rate; LVDP, left ventricular-developed pressure.

The levels of the heart rate and LVDP were immediately decreased upon the commencement of the ischemia and reached zero level during the ischemia in the groups exposed to the global ischemia. The values of the hemodynamic parameters obtained in the preconditioned and non-preconditioned hearts at different times of the preischemia and postischemia are shown in Table 1. As this table shows, there was an improvement in the hearts that underwent the RIPC with the values changed from 47.42 ± 6.27 mmHg in the sham-operated group to 85.22 ± 7.9 mmHg in the preconditioned group. The percentage of cardiac rate pressure produce (RPP%) as an indicator of heart performance, was significantly (*p* < 0.0001) declined in the I/R and sham-operated groups compared to the control group. However, the RPP% in the group exposed to the hepatic RIPC reached the same level as the control group. The effects of hepatic RIPC on the rate pressure of the isolated heart subjected to 30-min ischemia followed by 90-min reperfusion are shown in Figure 2.

**Table 1:** Values of the hemodynamic parameters in the non-preconditioned and preconditioned hearts are measured at the different phases of preischemia and postischemia.

	**Preischemic**	**Endischemia**	**Reperfusion time**				
			**2 min**	**15 min**	**30 min**	**60 min**	**90 min**
**HR**							
Control	218.53 ± 4.3	210.1 ± 7.5	214.46 ± 8.9^a^	214.96 ± 8.3	211.66 ± 10.1	201.5 ± 11.4	197.73 ± 14.2
Ischemia/Reperfusion(I/R)	243.96 ± 10.6	0^a^	242 ± 12.6	238.3 ± 11.7	230.33 ± 15.8	212.33 ± 6.4	212.66 ± 5.5
Sham	242.93 ± 12.1	0^a^	242.8 ± 18.6	222.02 ± 14.5	216.86 ± 18.2	216.93 ± 6.7	205.09 ± 5.67
Treatment	251.5 ± 6.6	0^a^	266.64 ± 17.84^a^	238.06 ± 17	244.73 ± 10.9	234.9 ± 8.1	230.73 ± 12.1
**LVDP**							
Control	89.1 ± 4.2	90.35 ± 7.1	89.19 ± 5.3	79.09 ± 9	86.68 ± 7	84.38 ± 7	82.85 ± 7.1
Ischemia/Reperfusion(I/R)	94.32 ± 1	0^a^	69.78 ± 3.7^a^	75.12 ± 5.9	76.41 ± 3.9	70.66 ± 2.7	65.26 ± 0.4^a^
Sham	78.12 ± 5.1	0^a^	64.27 ± 8.4^a^	56.13 ± 5^a^^,^^b^	58.14 ± 7.5^a^^,^^b^	53.9 ± 8.9^a^	49.75 ± 7^a^
Treatment	91.75 ± 2.8	0^a^	86.79 ± 2.6	96.18 ± 5.7^b^^,^^c^	97.04 ± 1.9^b^^,^^c^	93.19 ± 2.5^b^^,^^c^	90.15 ± 4.4^b^^,^^c^

*Data are expressed as means ± SE. Obtained from at least six tested mice in each group*.

*HR, heart rate (beats/min); I/R, ischemia/reperfusion; LVDP, left ventricular developed pressure (LV systolic minus LV diastolic pressure, mmHg)*.

a *p < 0.05 vs. control*.

b *p < 0.05 vs. I/R*.

c *p < 0.05 vs. sham-operated*.

**Figure 3:**
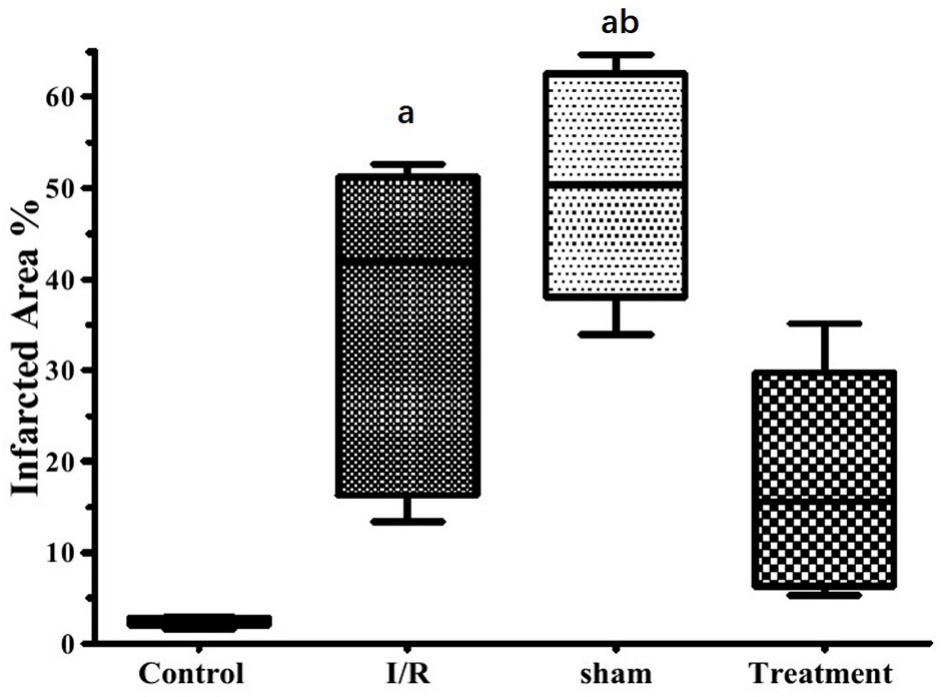
The effects of liver remote ischemic preconditioning on I/R1-induced myocardial infarct size in the isolated perfused rat hearts. Data are obtained from at least six tested mice in each group. a = *p* < 0.001 vs. control, b = *p* < 0.001 vs. treatment. I/R, ischemia/reperfusion.

**Figure 4:**
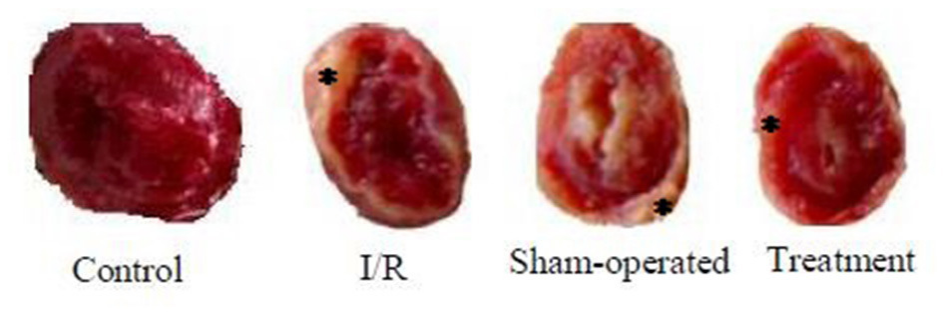
The representative images of the infarct size data were taken from control, I/R, sham-operated, and treatment groups. ^*^ denotes the myocardial scar. I/R, ischemia/reperfusion.

The effect of hepatic RIPC on I/R-induced myocardial infarct size in the isolated perfused hearts is shown in Figure 3. As this figure illustrates, the hearts exposed to 30-min ischemia followed by 90-min reperfusion showed a median infarcted area of 41.90 and 50.30% in both the I/R and sham groups, respectively. However, the isolated hearts preconditioned by hepatic intermittent periods of ischemia and reperfusion showed a significant (*p* < 0.001) reduction in infarct size to 15.60%. The representative images of the infarct size data are shown in Figure 4.

**Figure 5:**
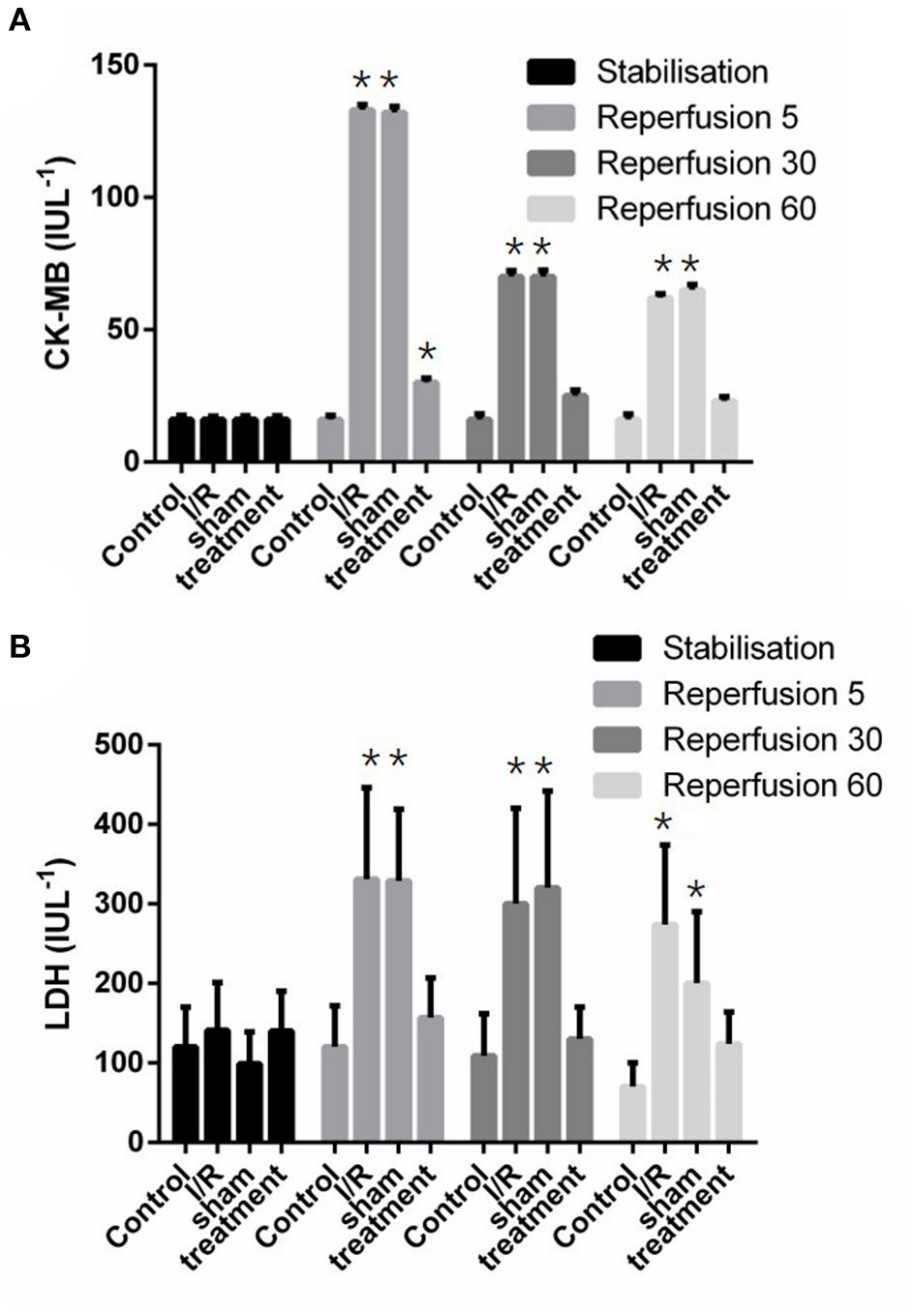
The effect of liver remote ischemic preconditioning on the release of CK-MB **(A)** and LDH **(B)** in the isolated rat heart subjected to 30-min global ischemia followed by 90-min reperfusion. Values are expressed as means ± SD obtained from at least six tested mice in each group. ^*^p < 0.001 vs. treatment and sham-operated group. CK-MB, creatine kinase-myocardial band; LDH, lactate dehydrogenase.

The release of cardiac LDH and CK-MB enzymes in different experimental groups is shown in Figure 5. This figure shows that significant (*p* < 0.001) increased enzyme releases were observed in the isolated perfused hearts subjected to I/R. However, there was significant (*p* < 0.001) reductions in the release of lactate dehydrogenase from 299.01 ± 10.7 to 152.3 ± 16.7 IUL^−1^ and phosphocreatine kinase-MB from 73.86 ± 1.95 to 25.93 ± 0.66 IUL^−1^ in the preconditioned group (Figure 5).

## Discussion

There is a body of evidence that suddenly the restoration of blood flow or reperfusion to the ischemic myocardium may paradoxically exacerbate the myocardial injury induced by ischemia (Piper et al., 1998). This aggravated damage, known as “reperfusion injury,” may lead to the additional injury and death of cells that are lightly injured during the preceding ischemic period. Numerous intervention procedures have been launched to attenuate the myocardial injury induced by I/R, wherein the RIPC is the newest ones. Recent investigations have revealed that the induction of intermittent ischemia in an organ far from the heart, referred to as RIPC, can limit heart damage following coronary occlusion and reperfusion (Przyklenk et al., 1993). In this study, we found that three episodes of short ischemia to the liver can lead to a significant reduction in myocardial damage and consequently an improvement in the performance of the hearts exposed to I/R. It suggests that the protective effect of hepatic RIPC can be remembered (Tokuno et al., 2002). It has been proposed that the humoral circulating cardioprotective factor released by RIPC and neuronal pathways may be related to the initiation and transfer of such signaling (Donato et al., 2021). However, the results of this study indicate that the signaling process may be memorized within the explanted heart, independent of ongoing neuronal stimulations. The survival improvement and decreased enzyme release associated with IPC have already been reported by Lioris-Carsi et al. in rat liver (Lloris-Carsi et al., 1993). In this study, it is found that the hepatic RIPC may protect the myocardium against I/R-induced injury once the heart is explanted. This cardioprotective effect was manifested by different indices of infarct size, the release of enzymes from the cardiac cells, and an improvement in the RPP%.

Following an acute myocardial infarction, temporary restoration of blood flow to the ischemic myocardium, either by thrombolytic therapy or *via* primary PCI, remains the main therapeutic strategy to limit the myocardial infarction or improve the cardiac performance. Owing to the correlation between the extent of infarcted area and the incidence of acute mortality, morbidity, and heart failure minimizing the size of the infarcted area is an important therapeutic goal to improve cardiac function.

The mechanism underlying myocardial cell death induced by I/R is still a matter of debate in the ongoing literature for some decades. Freude et al. reported that necrosis is the predominant cell-death pattern during the ischemia period. They found that apoptosis, another cell-death mechanism, was absent during ischemia and it was increased during the procedure of reperfusion (Freude et al., 2000). It suggests that the process of the injury and cell death occurs in both phases of ischemia and reperfusion, but the severity of the injury during reperfusion is further. A preventive strategy is also required to reduce the damage during both stages to improve the clinical symptoms in patients with acute myocardial infarction. As previously reported (Chen et al., 2018), RIPC-induced cardiac protection against I/R injury in the heart of the rat is correlated with the activation of antiapoptotic signaling. Cellier et al. documented that increased mitochondrial fusion protein OPA1 and preserved mitochondrial morphology could play an important role in RIPC-induced cardioprotection (Cellier et al., 2016). Short episodes of I/R from the remote site may stimulate circulating nitrite, which contributes to cardiac protection during I/R (Rassaf et al., 2014). The results of some studies in the past suggest that in some clinical conditions, the use of IPC can meet this necessity.

To avoid an invasive approach to the myocardium, an alternative strategy for IPC is the use of a remote organ far from the heart for induction of preconditioning in the myocardium tissues. In this study, the liver chosen as the remote organ was subjected to three cycles of 5-min ischemia by occlusion of the portal triad in anesthetized mice. The heart was then, excised and exposed to 30-min ischemia followed by 90-min reperfusion using a Langendorff system. Hepatic RIPC caused a significant reduction in RPP%, the cardiac infarct size, and myocardial enzymes (LDH and CK-MB). The current results suggest the cardioprotective effectiveness of liver RIPC against I/R-induced myocardial cell injury. Consistently, it has been reported that hepatic RIPC had significantly diminished the incidence of I/R-induced arrhythmias in isolated rat hearts (Noorbakhsh et al., 2015). Furthermore, Yang et al. reported that the hepatic RIPC protected the heart against I/R injury *via* GSK-3β-dependent cell-survival signaling pathway (Yang et al., 2017).

This study investigated the cardioprotective effects of liver RIPC against I/R-induced injury in isolated perfused rat hearts. It was found that three short episodes of hepatic ischemia can lead to a significant decrease in myocardial injury induced by I/R. The results of this study have provided evidence that surgery of an organ may have protective effects on other limbs, namely, the heart in deficiency conditions.

## Data Availability Statement

The raw data supporting the conclusions of this article will be made available by the authors, without undue reservation.

## Ethics Statement

The animal study was reviewed and approved by Ethics Committee of Beijing Anzhen Hospital.

## Author Contributions

YR and SL were responsible to the manuscript writing and equally contributed to this manuscript. WL was responsible to the data analysis. HD designed the whole study. All authors contributed to the article and approved the submitted version.

## Funding

This project was supported by grants from the Beijing Natural Science Foundation (No. 7184205), National Natural Science Foundation (81970525), Beijing Hospitals Authority Incubating Program (No. PZ2021007), and Beijing Hospitals Authority Youth Program (No. QML20200604). The sponsors had no role in the study design, data collection and analysis, decision to publish, or manuscript preparation.

## Conflict of Interest

The authors declare that the research was conducted in the absence of any commercial or financial relationships that could be construed as a potential conflict of interest.

## Publisher's Note

All claims expressed in this article are solely those of the authors and do not necessarily represent those of their affiliated organizations, or those of the publisher, the editors and the reviewers. Any product that may be evaluated in this article, or claim that may be made by its manufacturer, is not guaranteed or endorsed by the publisher.
